# PubMed Phrases, an open set of coherent phrases for searching biomedical literature

**DOI:** 10.1038/sdata.2018.104

**Published:** 2018-06-12

**Authors:** Sun Kim, Lana Yeganova, Donald C. Comeau, W. John Wilbur, Zhiyong Lu

**Affiliations:** 1 National Center for Biotechnology Information, National Library of Medicine, National Institutes of Health, Bethesda, Maryland 20894, USA

**Keywords:** Publication characteristics, Literature mining, Data mining

## Abstract

In biomedicine, key concepts are often expressed by multiple words (e.g., ‘zinc finger protein’). Previous work has shown treating a sequence of words as a meaningful unit, where applicable, is not only important for human understanding but also beneficial for automatic information seeking. Here we present a collection of *PubMed^®^ Phrases* that are beneficial for information retrieval and human comprehension. We define these phrases as coherent chunks that are logically connected. To collect the phrase set, we apply the hypergeometric test to detect segments of consecutive terms that are likely to appear together in PubMed. These text segments are then filtered using the BM25 ranking function to ensure that they are beneficial from an information retrieval perspective. Thus, we obtain a set of 705,915 *PubMed Phrases*. We evaluate the quality of the set by investigating PubMed user click data and manually annotating a sample of 500 randomly selected noun phrases. We also analyze and discuss the usage of these *PubMed Phrases* in literature search.

## Background & Summary

Unlike other general domains, the language of biomedicine uses its own terminology to describe scientific discoveries and applications. To understand the semantics of biomedical text, it is important to identify not only the meaning of individual words, but also of multi-word phrases appearing in text^1^. Finding phrases is a fundamental but often overlooked process. Controlled vocabularies such as dictionaries and ontologies may help, but maintaining those is costly and their coverage is known to be limited. As demonstrated in a study of PubMed search^2^, meaningful phrases represent a significant fraction of queries in PubMed. This suggests that users, in many cases, have phrase(s) in mind when entering a query, e.g., ‘*Central venous pressure*’ and ‘*familial Mediterranean fever*’. While PubMed search interprets these queries as a conjunction of individual terms, an earlier study^2^ demonstrates that there is a qualitative difference between the results containing all individual terms and those containing the phrase. Therefore, it would be beneficial to interpret such queries as phrases.

From a corpus linguistic point of view, coherent phrases are similar to collocations. A collocation is an expression consisting of two or more words that correspond to some conventional way of saying things^3^. This notion emphasizes the necessity of finding multi-word expressions in the biomedical domain. For example, a common colloquial expression such as ‘*flu shot*’ is rare in PubMed, while ‘*influenza vaccine*’ is the biomedical term used for the same concept. Our phrases and collocations share some similarities, but the main difference comes from grammatical completeness. Collocations are restricted to noun/adjective phrases or phrasal verbs, whereas we do not limit phrases grammatically, but rather see them as more flexible entities to be used as building blocks to form longer phrases or sentences. Such an interpretation of phrases is better aligned with our goal of using the corpus to analyze queries, as queries may frequently contain incomplete phrases and, in general, are known to differ from traditional forms of written language^4^. This makes us believe that statistical methods are better suited for our goal.

While the task of identifying the most useful phrases has been studied extensively^1,5–9^, it remains challenging. Several studies have relied on natural language parsers to extract well-formed phrases such as noun, verb and/or prepositional phrases^5,7,8^. Other studies using statistical methods include noun phrase query segmentation^10^ and well-formed phrase extraction from MEDLINE^®6^. Another work developed for multi-word expression part-of-speech (POS) tagging^11^ utilizes neural networks to identify phrases. However, all the statistical approaches mentioned above are supervised, and thus require training sets to achieve their goal. Furthermore, it is unknown whether the results would improve search performance.

Our approach is unsupervised, guided first by the hypergeometric test to determine whether a sequence of words is a coherent segment. The strings that pass the hypergeometric test are further treated as queries and evaluated in terms of their effectiveness in document retrieval in a comparative way: by treating them as a phrase versus as individual words. For example, given an extracted phrase ‘*lung cancer patients*’, we would compare retrieval performance when treating it as ‘*lung cancer patients*’ or as individual words, ‘*lung*’, ‘*cancer*’ and ‘*patients*’. This step is designed to select those phrases which result in improved information retrieval performance. Here, we use the BM25 ranking function^12^ for retrieval.

To compute and compare the retrieval performance in the absence of a manually annotated gold standard, we use a novel pseudo-relevance judgement technique, which is based on the assumption that the documents containing query terms in the titles are more relevant to the query than the documents that do not^13^. Guided by this evaluation, we collect a set of 705,915 multi-word strings that benefit from being interpreted as phrases rather than individual tokens in terms of retrieval performance. We refer to this set as *PubMed Phrases*. *PubMed Phrases*, as an open data collection, represents a rich knowledge base of over seven hundred thousand phrases available to the scientific community. The resource can be readily used for text mining tasks including query/text segmentation, document understanding and beyond. Note that, throughout this paper, the term *phrase* refers to a coherent chunk of words that are frequently used together.

## Methods

**Figure 1:**
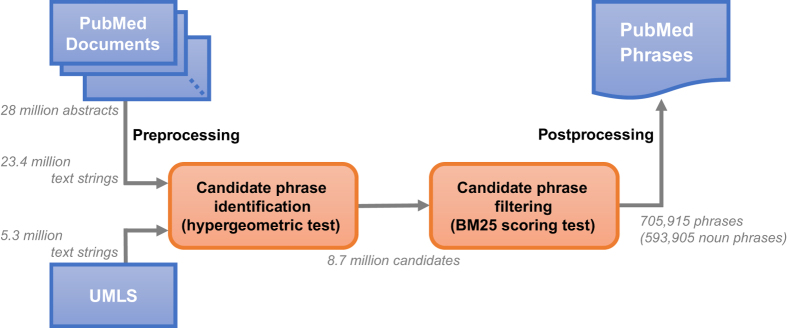
Workflow of *PubMed Phrase* extraction.

In this section, we present our unsupervised framework for computing *PubMed Phrases*. This method consists of two steps: 1) obtaining candidate phrases from PubMed titles/abstracts and UMLS based on the hypergeometric test and 2) filtering the phrases using the BM25 ranking function. The first step segments text strings from PubMed documents and selects those segments that are likely to be used as a unit. The second step examines the phrases further to validate whether using the candidate as a coherent unit benefits retrieval performance. Figure 1 depicts an overall workflow of our approach.

### Identifying candidate phrases

We start by preprocessing the entire PubMed and compiling a comprehensive list of multi-token text segments from the literature. From titles and abstracts of PubMed documents we collect all multi-word text strings that are bounded by punctuation or stopwords, and appear at least 5 times in PubMed. For this process, hyphens and apostrophes are not used as delimiters. As of late 2017, this resulted in 23.4 million unique text strings. This approach limits our space to phrases without prepositions or other stopwords. It has been shown that the majority of meaningful biomedical phrases that appear in both UMLS^®^ and PubMed do not include stopwords^1^. However, to avoid missing meaningful phrases with stopwords, we also extracted 5.3 million phrases from UMLS and added them as candidates. This allows to identify some good quality phrases such as ‘activin a’.

Next, we process the multi-word strings to identify substrings that are observed in the PubMed literature as coherent units. Given two words, *word*_*1*_ and *word*_*2*_, the *p*-value is computed to determine whether they appear together in PubMed by chance. Let *N*_*s*_ be the number of sentences that contain *word*_*1*_, *N*_*t*_ be the number of sentences that contain *word*_*2*_, *N*_*st*_ be the number of sentences that contain the words *word*_*1*_ and *word*_*2*_, and *N* be the total number of sentences in PubMed. The random variable *Y* representing the number of sentences containing *word*_*1*_ and *word*_*2*_ is a hypergeometric random variable with parameters *N*_*s*_, *N*_*t*_ and *N*^14^. The probability distribution of *Y* is shown as follows:

$$P\left(Y=y\right)=\frac{\left(\begin{array}{c} N_{t}\\ y \end{array}\right)\left(\begin{array}{c} N-N_{t}\\ N_{s}-y \end{array}\right)}{\left(\begin{array}{c} N\\ N_{s} \end{array}\right)}$$

From *N*_*st*_ we compute the *p*-value, i.e., the probability of the observed *N*_*st*_ or a higher frequency arising by chance as follows:

$$p-\mathrm{value}=\sum_{y=N_{st}}^{\min\left(N_{s},\;N_{t}\right)}P\left(y\right).$$

Since our interest is in computing whether *word*_*1*_ and *word*_*2*_ form a phrase, we place a more stringent requirement on the hypergeometric test. We define *N’*_*st*_ as the number of sentences that contain the phrase *word*_*1*_ *word*_*2*_ (a subset of *N*_*st*_) and replace the value of *N*_*st*_ with *N’*_*st*_ when we compute the probability *P*(*y*) and the corresponding *p*-value. If the observed frequency of a phrase is above the expected value for a random incident (we set the *p*-value threshold to 0.01), we conclude that *word*_*1*_ and *word*_*2*_ form a coherent segment and retain this segment for further processing.

**Figure 2:**
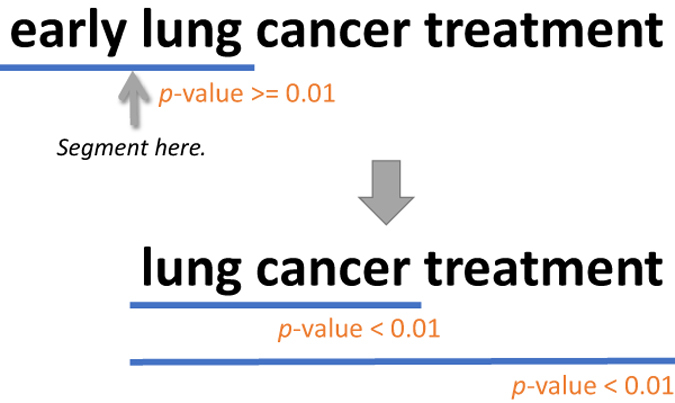
Example of text segmentation using the hypergeometric test.

In this work, we also expand the *p*-value test by replacing *word*_*1*_ with a text segment, i.e., the hypergeometric distribution is used to test the significance of a text segment and a word appearing together. Once a pair of words has been pooled, the algorithm considers the relationship between the segment *word*_*1*_ *word*_*2*_ and the next token *word*_*3*_, by computing the *p*-value score between *word*_*1*_ *word*_*2*_ and *word*_*3*_. The process continues until we either reach the end of the multi-word text string (we collect these strings) or encounter a token *word*_*i*_ that does not pass the *p*-value test when tested against the chunk *word*_*1*_…*word*_*i-1*_ (we discard these strings). Figure 2 shows an example of how text is segmented using the hypergeometric test. In the figure, the statistics for terms ‘*early*’ and ‘*lung*’ suggests that they appear together by chance, but ‘*lung cancer treatment*’ appears as a unit in PubMed and it is statistically significant.

Since we perform multiple hypergeometric tests for each candidate, we adopt a useful statistical correction for multiple comparisons, the Benjamini-Hochberg correction^15^, which allows one to achieve the desired confidence level for a phrase when multiple comparisons are performed. Using the Benjamini-Hochberg correction, we eliminated 11 phrases that do not pass the set *p*-value threshold. As a result, we obtained 8,730,788 candidates from 28.7 million multi-word strings in this first step. We do not apply a multiple testing correction to this set but rather accept a 1% error rate in the 8.7 million candidates.

Traditional approaches of identifying collocations are mutual information, log likelihood or the chi-square test^16–18^. In particular, it has been observed that the hypergeometric test and mutual information provide similar performance^19^. However, for mutual information, one must set a threshold to determine the significance of co-occurrence of two words. The threshold varies from corpus to corpus, and does not offer an intuitive probabilistic interpretation. For the hypergeometric test one must also set a *p*-value threshold, but the *p*-value of 0.01 is a widely accepted standard for significance, while there is no such standard for mutual information. Furthermore, the hypergeometric test returns a value that directly indicates the statistical significance of an observed event.

### Evaluating phrases for their effectiveness in document retrieval

The resulting candidates obtained from the previous step are statistically significant. However, there is no guarantee that treating these text segments as phrases would improve the performance of document retrieval. To address this issue, we apply a simple but effective procedure that we call the BM25 scoring framework.

The BM25 scoring framework is designed to detect those text segments that benefit the search when interpreted as phrases. This process is optimized towards retrieval performance and guarantees that each selected phrase is a coherent unit and improves the retrieval performance when treating the phrase as a unit. However, a difficulty with this approach is that the evaluation of retrieval results requires a significant effort in human judgements. To address this problem, we employ an automatic approach to efficiently compare search results without manually creating a gold standard set.

Our method is based on the following hypothesis:

As a class, the documents that do not contain all query terms in the title, but do contain all in the abstract (set A) qualitatively differ from the records that do contain all the query terms in the title and all in the abstract (set T). The difference between set A and set T reflects the characteristics of these two sets that make records in T more likely than records in A to be useful to an information seeker.

**Figure 3:**
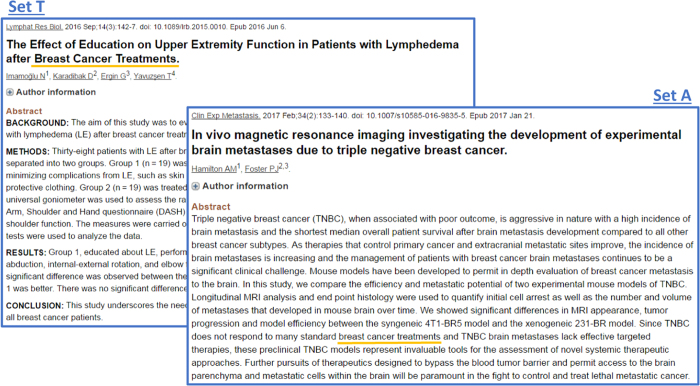
Comparison of a document including the phrase, ‘*breast cancer treatments*’ in the title versus a document including the same phrase in the abstract.

There are two reasons why this hypothesis is highly plausible. First, we believe that titles are carefully chosen to be good indicators of the contents of scientific articles. Second, studies analyzing user behavior in response to PubMed retrieval^20^ have found that an article was more likely to be clicked on if the query terms appeared in the title. If we use a likelihood of being clicked as a pseudo-relevance measure, it justifies our hypothesis that documents with query words in a title are likely to be more relevant than those documents that contain query words only in the abstract. Figure 3 illustrates our hypothesis with two documents, each containing the phrase ‘*breast cancer treatments*’. One has the phrase in the title (set T) and the other has the same phrase in the abstract (set A). Both are related to ‘*breast cancer treatments*’ however it is clear the one belonging to set T is more relevant to the query ‘*breast cancer treatments*’. We further discuss the validity of the hypothesis in Technical Validation.

For more precise evaluation, synonymous words/phrases should be taken into account to judge the relevance of documents. While this complex approach would work better, it may raise the ambiguity problem among synonymous phrases. Thus, we use a simple approach relying only on exact word/phrase matching and believe that it should provide a reasonable metric for detecting useful phrases.

Each phrase candidate identified by the hypergeometric test is now used as a query. We first create the appropriate set A∪T for the phrase. We then re-rank these abstracts based on BM25 scores for two scenarios: 1) The traditional way treating the query as represented by its individual words, which scores the abstracts based on the sum of the weights of individual words and 2) Computing BM25 scores by treating a query (or phrase) as a single multi-word term, not using the individual words in the scoring.

The intuition behind this comparison can be seen from a simple example. Consider the phrase ‘*zinc finger*’ and consider the individual words, ‘*zinc*’ and ‘*finger*’. If ‘*zinc*’ and ‘*finger*’ appear in a sentence or a title, it is very likely they would appear as the phrase ‘*zinc finger*’. If ‘*zinc*’ and ‘*finger*’ occur in an abstract, but not as the phrase ‘*zinc finger*’, it is highly unlikely the phrase ‘*zinc finger*’ will appear in the abstract or the title. Thus, that document will have been marked as a negative and also will not be retrieved by the phrase retrieval, but will be retrieved by the usual individual word retrieval. It is in this situation that we can see the phrase retrieval outperform the individual word retrieval. This also illustrates why the phrase retrieval can benefit the user. If the user is interested in ‘*zinc finger*’, they will not likely be interested in documents that mention ‘*zinc*’ and ‘*finger*’ separately.

In our framework, BM25 ranking of candidate documents is performed using only the abstracts of PubMed documents. Titles are used only to assign positive or negative labels. A document is labeled as *positive* (set T) if its title contains all query tokens, and *negative* (set A) otherwise. In the BM25 scoring framework, we measure the retrieval performance using average precision^21^, i.e., the average of precisions across all ranks containing relevant documents.

We collected *PubMed Phrases* following the four criteria:

1. For every candidate phrase, a set of titles containing all individual words in a phrase and a set of abstracts containing the same words should have at least five documents in common.
2. The search performance should be higher when a query is treated as a phrase as compared to being treated as individual query words.
3. The search performance of a phrase in terms of average precision should be higher than the baseline performance, which is the score obtained when a set of documents is randomly ranked.
4. The average precision using the BM25 individual word retrieval must be higher than 0.01. A lower average precision from the search may mean there is insufficient evidence for judging relevance in the set, thus we discard such phrases.

Filtering the candidate phrases to satisfy the four criteria above resulted in 705,915 *PubMed Phrases*. If we limit the lower bound of performance improvement to 10% in criterion 2, we obtain the subset of 568,125 phrases that we will refer to as *PubMed*_*small*_.

## Data Records

The *PubMed Phrase* set is a collection of 705,915 coherent text segments that improve retrieval performance when interpreted as phrases rather than individual words. An interesting property of the *PubMed Phrase* set is that it is computed using completely data-driven approaches without considering POS tags. As a result, the set includes more data than the traditionally favored well-formed noun phrases. We examined the composition of the set and found that 84.1% of the phrases are noun phrases. The remaining 15.9% of phrases are frequently used coherent segments that are found to improve the retrieval performance despite not being noun phrases such as ‘*high resolution 3d*’.

While the statistical filtering step using the hypergeometric test focuses on the likelihood of words appearing as a unit, the empirical filtering using BM25 analyzes how PubMed authors prefer to refer to a concept. For example, the candidates that pass the hypergeometric test but not BM25 include ‘*cavoatrial tumor*’ and ‘*Kentucky farmers*’. While these phrases look reasonable, ‘*cavoatrial … tumor thrombus*’ and ‘*farmers* … *in Kentucky*’ are also commonly used to express the same concepts.

Table 1 shows the evaluation results of *PubMed Phrases* using the BM25 scoring framework. The performance of the selected *PubMed Phrase* set achieves significantly higher performance in phrase-based search for both *PubMed*_*all*_ and *PubMed*_*small*_ sets.

The 705,915 multi-word strings of the *PubMed Phrase* set can be found in ‘all_dictionary.txt’ [Data Citation 1] (Future updates of the *PubMed Phrase* set may be available at https://www.ncbi.nlm.nih.gov/research/bionlp/data). There are four additional datasets released with the list of *PubMed Phrases*. The first set (‘all_dictionary.pos’) includes POS tags of each phrase. Since POS tags used can differ depending on context, we extracted the two most common POS tags appearing in PubMed abstracts and included them in the POS tag file. The second set (‘all_dictionary.group’) clusters the *PubMed Phrase* set by assessing whether a phrase contains other phrases in the set. Each line starts with a phrase and is followed by substrings that also appear in the *PubMed Phrase* set. For example, the phrase, ‘*super heavy oil*’, includes two phrases ‘*heavy oil*’ and ‘*super heavy*’ from the same set. The third set (‘all_dictionary.pmid’) includes all PMIDs containing a phrase, for each phrase. Finally, the fourth set (‘all_dictionary.sco’) is a list of vertical bar separated lines, where each line contains a phrase in the first column, a set of *p*-value scores for every subphrase considered in the second column, and two average precision scores in the third column. The first average precision score is based on the BM25 ranking treating each word individually, while the second by treating the phrase as a single unit. For example, given the phrase ‘*natural language processing*’, the second column contains *p*-value scores for *natural*/*language* and *natural language*/*processing*. The third column reports the BM25 scores of search treating the phrase as individual words, ‘*natural*’, ‘*language*’ *and* ‘*processing*’ versus as a phrase, ‘*natural language processing*’. The POS tag, group, PubMed ID, and score files provide a wealth of information that could be useful for analyzing and selecting a subset of *PubMed Phrases*.

## Technical Validation

### Validity of using PubMed titles for relevance judgment

Our relevance measure for the phrase filtering step assumes that a document with title containing all query terms is likely to be relevant to the user issuing the query (i.e., phrase). Titles in scientific documents are normally chosen to provide a good summary of the article^13^. Therefore, it is logical to assume that a document is more likely relevant to a user issuing a query if its title contains all query terms. This is supported by the empirical observation that users prefer to click on documents containing query terms in the title^22^. We further noticed from the PubMed user logs that, given documents scored by BM25, users are four times more likely to click on a document containing query terms in the title than on a document that does not. This conclusion is further bolstered by several studies documenting that user clicks are strongly correlated with relevance^23–25^. Note that we are not making any assertion about the documents that contain query terms in the abstract and not in the title, as a number of them may also be relevant. Moreover, it is less obvious how to judge relevance of a document whose title contains some of the query terms but not all. Therefore, we take a more conservative approach and treat those documents as negative for computing average precision. As a consequence, the average precision we compute is only an approximate lower bound for the true average precision, but when this lower bound is quite high we believe that is evidence that supports our argument.

BM25 is one of the popular ranking functions and known to be a good performer in document search^12^. In PubMed, BM25 can be applied to both titles and abstracts for search. However, in our experiments, we apply BM25 to abstracts only, and use titles as the indicator for relevance. Here is the logic of our arguments to use PubMed titles for relevance judgments:

1. BM25 provides good performance in retrieving relevant documents.
2. If we assign positive labels to those documents that have all query words in the titles and evaluate BM25 retrieval based on that assignment, we find that BM25 produces average precisions that are significantly higher than a random ranking.
3. The obvious explanation for #2 is that positive labels are being assigned preferentially to the most relevant documents.

We believe 1-3 above provide significant support for our assumption that query words in the title are a useful indication of relevance. To experimentally validate item 2 here, we chose 27,870 frequent user queries from PubMed^26^, and performed BM25 ranking for all abstracts with query words in them. This was then compared with a random order of the same PubMed document set for each query. Using the criterion that documents having the query words in the title are counted as relevant, BM25 achieved 0.3490 average precision, whereas the random document ranking achieved 0.1759 average precision. The difference is substantial and it shows that using titles to create a relevance standard is a practically sound strategy.

### User click-based evaluation of *PubMed Phrases*

Although there are various factors to affect user clicks, a consensus of user clicks may provide a clue of how useful *PubMed Phrases* are. Therefore, we performed a user click-based experiment as follows. We first chose the 100 *PubMed Phrases* that were also the most frequent queries in 2017. Assuming the document that is clicked is relevant to the query, we examined the top 20 retrieved documents from all PubMed users. 20 is the default number of documents displayed in PubMed on the first page. For each query, we compared click rates between two groups of documents: 1) the documents with query terms appearing as the phrase and 2) the documents with query terms not appearing as the phrase. By click rate of a given set of documents, we mean the fraction of the documents that are clicked. We computed the click rate difference for each query, and averaged these differences over the 100 *PubMed Phrases* under consideration. The results show that, when a query appears as the phrase in a document, the click rate is 96.5% higher than when it does not appear as the phrase, on average. Four of these 100 queries have lower click rates when the phrase is present, however, these four cases have very few documents that have the query terms but not the phrase, making these cases statistically unreliable. This means using the *PubMed Phrases* as phrases has the potential to benefit PubMed search.

### Manual evaluation of *PubMed Phrases*

To assess whether phrases in the *PubMed Phrase* set satisfied criteria of well-formedness and completeness, we conducted manual evaluation of a randomly selected subset of phrases. The annotation task was performed in two rounds and conducted by two annotators with backgrounds in biomedical informatics research and experience in annotating biomedical corpora.

**Figure 4:**
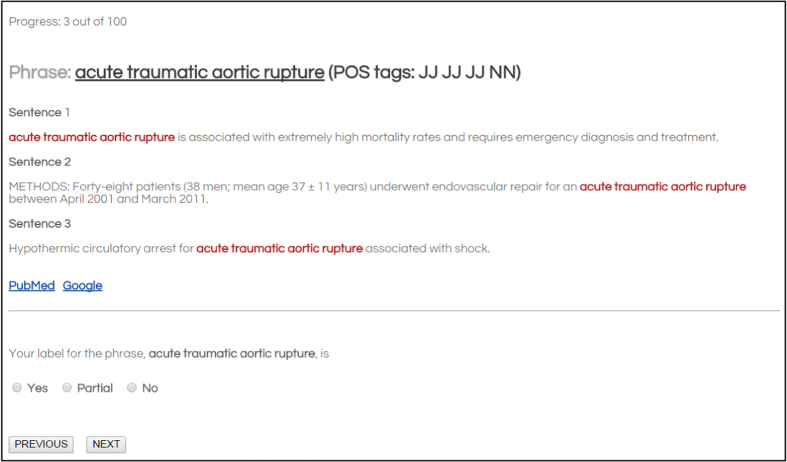
Manual annotation interface for *PubMed Phrases*. Each annotator assigned yes (positive), no (negative) or partial by reviewing a given phrase and three PubMed sentences including the phrase.

During a preliminary round, we observed that human annotators naturally preferred well-formed noun phrases and it was challenging to come up with clear annotation guidelines for annotating non-noun phrases. For that reason, we decided to sample for manual annotation five hundred noun phrases by considering POS tags and choosing phrases ending in singular noun (NN) or plural noun (NNS) POS tags. In the first round, each annotator was given a phrase and three sample sentences containing the phrase and asked to assign one of the three labels: *positive*, *negative* and *partial*. Figure 4 shows the web interface we used for manual evaluation.

The annotators were asked to keep the following questions in mind when assigning a label to a phrase:

- Does a phrase have a clear meaning on its own?
- Is a phrase obviously missing terms?

A phrase was labeled *positive* if it had a specific meaning and was a complete noun phrase. A phrase was labeled *negative* if it was judged not to be a phrase but a collection of terms. Finally, a phrase was labeled *partial* if it had a specific meaning but was missing certain terms.

After the first round of evaluation, the annotators had agreed upon 476 phrases (95.2% inter-annotator agreement), assigning 463 phrases as *positive* and 13 phrases as *partial*. Examples with the *partial* labels are ‘*locus yac*’ and ‘*mushroom macrolepiota procera*’. The ‘*locus yac*’ was judged partial because in the example sentences, it was always used with ‘*beta-globin*’, as ‘*beta-globin locus yac*’. The meaning of ‘*mushroom macrolepiota procera*’ is quite specific, but usually it is referred to as ‘*parasol mushroom*’ or ‘*macrolepiota procera*’.

During the second round, the annotators were asked to review the phrases for which they had not achieved agreement in the first round. After this stage the annotators had reached an agreement for all 500 phrases, of which 480 phrases were labeled *positive*, 3 phrases were labeled *negative* and 17 phrases *partial*. Four additional *partial* cases, found during the second round of annotations, are ‘*agri sp*’, ‘*iib myosin heavy chain*’, ‘*rat skeletal l6*’ and ‘*vivo footprints*’. ‘*rat skeletal l6*’ is typically used in the form ‘*rat skeletal l6*’ + *myotubes*/*myoblasts*/*cells*, while other phrases are missing certain words, e.g., ‘*sphingomonas agri sp nov*’ for ‘*agri sp*’, ‘*type iib myosin heavy chain*’ for ‘*iib myosin heavy chain*’ and ‘*in vivo footprints*’ for ‘*vivo footprints*’.

The three examples annotated as *negative* were ‘*coated multiparticulate*’, ‘*desert spiny*’ and ‘*immunoreactive activin*’. ‘*coated multiparticulate*’ is an adjective and usually followed by noun(s) such as ‘*systems*’ but the POS tagger assigned noun for ‘*multiparticulate*’. The full noun phrase of ‘*desert spiny*’ is ‘*desert spiny lizards*’ in general, but ‘*desert spiny*’ itself is a vague term. The most common phrase in PubMed that includes ‘*immunoreactive activin*’ is ‘*immunoreactive activin a*’, but ‘*a*’ was treated as a stopword in our process and the same term does not appear in UMLS (Note: ‘activin a’ is in our *PubMed Phrase* set). Moreover, ‘*a*’ is not the only noun that can follow the ‘*immunoreactive activin*’ segment. Although ‘*immunoreactive activin*’ is labeled as negative, it is still useful as a coherent chunk for improved literature search. The random phrases chosen for annotation and their evaluation results are available in the Supplementary Data.

## Usage Notes

While our main goal is to obtain a large collection of phrases from PubMed that can be beneficial for literature search, the collection of phrases can be used for other text mining and complex text-based tasks. For instance, document summarization methods^27,28^ frequently depend on mapping free text to a set of phrases and use these phrases as atomic units to generate summaries. Phrase-based statistical machine translation and paraphrase methods^29,30^ rely on phrase information and bilingual phrase tables. Document clustering tasks^31–33^ have been demonstrated to benefit from using phrase information. Using phrases as atomic search units has also been shown to improve the retrieval performance in a question answering task^34^.

Since 2017, the *PubMed Phrase* set has been used for indexing documents in PubMed’s new relevance search (https://www.nlm.nih.gov/pubs/techbull/jf17/jf17_pm_best_match_sort.html). In addition, we present two use cases to illustrate the utility of the set.

### Phrase Clouds versus Word Clouds for better knowledge visualization

**Figure 5:**
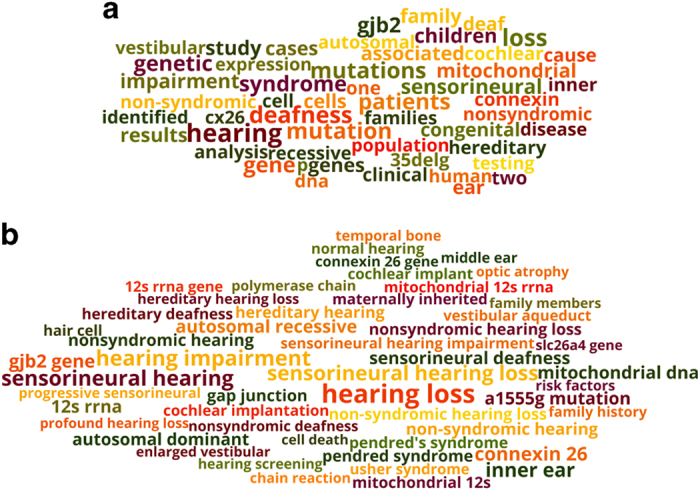
Tag clouds for the disease set, ‘*deafness*’. The clouds are based on top 50 single words (**a**) and top 50 *PubMed Phrases* (**b**) appearing in the set. Each term was weighted by the log of frequency.

Word clouds (or tag clouds) are widely used as a visualization tool that conveniently summarizes textual content. Word clouds are constructed to represent term size proportional to frequency, and they are typically based on single terms. With the availability of *PubMed Phrases*, an attractive alternative is to construct a word cloud based on phrases. Figures 5a and b compare the word clouds generated by single words versus *PubMed Phrases*. Both tag clouds are drawn from the same set of PubMed documents that represent ‘*deafness*’^28^. In Fig. 5a, single words are extracted from the set and weighted by the log of their counts. Figure 5b depicts a word cloud that is based on the phrases available in our *PubMed Phrase* set. The tag clouds are for the top 50 single words and top 50 phrases. We find that phrase clouds provide a more descriptive and user-friendly representation of the document set.

### Title generation in clusters

Clustering is a task of grouping a set of documents in such a way that documents with a similar topic belong to the same group. Clustering is usually an unsupervised process which computes clusters that are not self-descriptive, and one should further sort through the documents to understand the theme of a cluster. Presenting visual cues, such as cluster titles, can significantly improve the user perception of clustering results^35,36^. One way of describing the subject of a cluster is to select a few keywords or a sentence to represent a cluster^37,38^. Another way to approach the problem is to apply topic modeling techniques, such as Latent Dirichlet Allocation (LDA)^39^, which provides a list of topic terms for each topic it finds. Here we show an example of how to select descriptive titles from *PubMed Phrases* based on topic terms generated by LDA.

In the experiment, we used a subset of PubMed documents that represent ‘*cystic fibrosis*’, ‘*deafness*’, ‘*autism*’ and ‘*hypertrophic cardiomyopathy*’ from the OMIM disease set^28^. We ran LDA on this OMIM set using the gensim toolkit^40^. The number of topics was set to 10, and default parameters were used without further optimization. Our goal is, for each topic, to select representative phrases from the *PubMed Phrase* set using top 20 topic terms from LDA. To achieve this, we extracted all noun *PubMed Phrases* that match with the topic terms. We eliminated phrases that were substrings of longer phrases. Table 2 shows the topic terms and corresponding phrases for certain clusters in the ‘*cystic fibrosis*’, ‘*deafness*’, ‘*autism*’ and ‘*hypertrophic cardiomyopathy*’ data sets. We find suggested phrases to be reasonable and representative of the given topic single terms. In addition, as supported in the literature^36^, we find them clearer and easier to comprehend compared to single topic terms. Note that ‘*two cases*’ in the table is an artifact emerging from ‘*two*’ and ‘*cases*’ in the LDA topic terms. This is simply a consequence of the fact that LDA includes all terms in its analysis.

## Additional information

**How to cite this article**: Kim, S. *et al*, PubMed Phrases, an open set of coherent phrases for searching biomedical literature. *Sci. Data* 5:180104 doi: 10.1038/sdata.2018.104 (2018).

**Publisher**’**s note**: Springer Nature remains neutral with regard to jurisdictional claims in published maps and institutional affiliations.

## Supplementary Material



Supplementary Information

## Figures and Tables

**Table 1:** Number of phrases and mean average precision performance for individual word-based and phrase-based document retrieval.

	**Number of phrases**	**Mean average precision**	
		**Word-based retrieval**	**Phrase-based retrieval**
*PubMed*_*all*_	705,915	0.1967	0.2681 (+36.3%)
*PubMed*_*small*_	568,125	0.1713	0.2565 (+49.7%)
PubMed_all_ means the set following guidelines outlined in the previous section, i.e., the whole *PubMed Phrase* set. PubMed_small_ includes the phrases that improve search performance by a minimum of 10%.			

**Table 2:** Examples of topic terms and their corresponding noun phrases from *PubMed Phrases*.

**Topic terms from LDA**	**Phrases from** ***PubMed Phrases***
cystic, fibrosis, cf, patients, mutations, screening, cftr, gene, disease, mutation, diagnosis, clinical, pancreatic, genetic, one, carrier, pancreatitis, testing, two, associated	carrier screening, carrier testing, cf patients, cftr gene, cystic fibrosis gene carrier, disease-associated mutations
gene, deafness, mutations, mutation, cx26, hearing, loss, cells, genetic, connexin, gjb2, syndrome, 35delg, recessive, human, cases, congenital, two, children, protein	cx26 gene mutations, gjb2 gene, hearing loss, mutations 35delg, two cases
syndrome, x, autism, disorders, fragile, disorder, genetic, gene, mental, retardation, patients, developmental, clinical, chromosome, genes, study, autistic, associated, children, behavioral	autistic disorder, developmental disorders, fragile x chromosome syndrome, fragile x mental retardation gene, fragile x syndrome, fragile x-associated disorders, fragile-x mental retardation syndrome, mental disorder
mutations, cardiomyopathy, hypertrophic, hcm, cardiac, mutation, patients, gene, myosin, protein, familial, disease, chain, heart, genetic, genes, c, human, results, troponin	cardiac myosin, cardiac troponin c, familial hypertrophic cardiomyopathy mutations, heart disease
